# Supplementary material for: The clinical significance of inflammatory biomarkers, IL6 cytokine, and systemic immune inflammatory index in rabbit model of acute and chronic Methicillin-resistant Staphylococcus epidermidis-induced osteomyelitis
Source: PLoS One. 2024 Aug 29;19(8):e0309145. doi: 10.1371/journal.pone.0309145 (PMC11361425; doi:10.1371/journal.pone.0309145)
Supplement: Attachment — Submitted filename: PONE-D-24-02543.docx [file pone.0309145.s001.docx]

1- In line 203, the selection of sampling days for hematological tests is based on what and how?
2- In line 213, What sample was used in interleukin 6 assay?
3- In line 213, What was the measurement method, type and brand of the kit used to measure interleukin 6?
